# Illustrated Anatomy of a Rare Variant: Moynihan’s Hump With Independent Anterior and Posterior Cystic Arteries

**DOI:** 10.7759/cureus.105454

**Published:** 2026-03-18

**Authors:** Noah R Mitchell, Mark Sauceda-Izbrand, Soham Apte, Uchenna Uduma, Ian Kania, Abayomi G Afolabi, Les Keniston

**Affiliations:** 1 Department of Anatomy, University of Pikeville - Kentucky College of Osteopathic Medicine, Pikeville, USA

**Keywords:** anatomic imaging, caterpillar hump variation, cystic artery, cystic artery variation, dual cystic artery, moynihan’s hump (caterpillar hump)

## Abstract

Anatomical variation within Calot’s triangle can complicate hepatobiliary surgery and increase the risk of vascular injury during laparoscopic cholecystectomy (LC). Among these variations, a tortuous right hepatic artery (RHA), known as Moynihan’s hump, and the presence of dual cystic arteries have each been described individually; however, their concurrent occurrence is rarely documented and sparsely illustrated in the literature. During routine cadaveric dissection at the University of Pikeville - Kentucky College of Osteopathic Medicine (UP-KYCOM), a rare vascular configuration was identified, in which a Moynihan’s hump of the RHA gave rise to two independent cystic arteries supplying the gallbladder. Both branches originated near the Moynihan’s hump and entered Calot’s triangle separately. This report provides high-resolution cadaveric photographs and an original anatomical illustration that clarify the spatial relationships between the tortuous RHA and the dual cystic arteries. The combined visual documentation offers a practical reference for understanding how this variant may obscure normal surgical landmarks and increase the risk of misidentification during cholecystectomy. By presenting clear cadaveric visualization alongside an educational schematic, this case contributes to the limited literature describing the coexistence of Moynihan’s hump with dual cystic arteries and emphasizes the importance of incorporating anatomical variation into surgical training and anatomical education.

## Introduction

The gallbladder receives its arterial supply from the cystic artery, which most commonly arises from the right hepatic artery (RHA) within Calot’s triangle, the space bounded by the cystic duct, common hepatic duct, and inferior surface of the liver [1-5]. Although this pattern is considered typical, variations in the course and branching of the RHA and cystic artery are frequent and hold significant surgical relevance. Awareness of these anomalies is essential to prevent vascular injury during hepatobiliary procedures, particularly laparoscopic cholecystectomy (LC) [6-10].

One notable variation is Moynihan’s hump, also known as the caterpillar configuration, in which the RHA becomes tortuous as it courses near the gallbladder before entering the right hepatic lobe [3,6-10]. This close relationship between the RHA, cystic duct, and cystic artery can obscure normal landmarks in Calot’s triangle, increasing the risk of accidental RHA ligation and subsequent hepatic ischemia [3,6-10]. The reported incidence of Moynihan’s hump among surgical patients in a recent meta-analysis was 3.1% [3].

A second vascular anomaly of importance is the double cystic artery, in which separate anterior and posterior branches arise independently from the RHA rather than from a common trunk [2,8]. The prevalence of this variation ranges from 2% to 15%, with a pooled mean of 8.9% across nearly 10,000 cases [2]. Failure to identify both branches intraoperatively can result in incomplete ligation, hemorrhage, or iatrogenic injury to adjacent biliary and vascular structures [2].

While both Moynihan’s hump and the double cystic artery have been well documented individually, their concurrent presence remains uncommon. A previous cadaveric report by Kamath.B described a similar coexistence and published one of the few available photographs illustrating this variation [4]. Nevertheless, such combined anomalies remain underrepresented in the literature, with limited visual documentation and few detailed anatomical descriptions available. The present case adds to this scarce body of evidence by providing high-resolution cadaveric photographs and an original anatomical illustration that clearly demonstrate the simultaneous presence of Moynihan’s hump and dual cystic arteries. Beyond its surgical relevance, this finding underscores the importance of recognizing vascular variability within Calot’s triangle and supports the continued integration of such anatomical variations into surgical and medical education.

## Case presentation

A routine cadaveric dissection of the upper abdomen was performed on a 63-year-old male cadaver obtained through the Anatomical Gift Program (Dayton, OH, USA) for use in gross anatomy education. The specimen had been embalmed within 24 hours of death using a formalin-based fixative solution. The cadaver was positioned supine with the anterior abdominal wall previously reflected. Dissection focused on the hepatoduodenal ligament and the vascular structures of the porta hepatis.

To initiate exposure, the liver and diaphragm were gently elevated superiorly to provide improved access to the lesser omentum [11]. A strip of white paper was inserted into the omental foramen to enhance contrast and facilitate identification of deep structures [11]. Blunt dissection was employed to open the peritoneum overlying the hepatoduodenal ligament, revealing the components of the portal triad: the common bile duct laterally, the hepatic artery proper medially, and the hepatic portal vein posteriorly [11].

The common bile duct was traced superiorly to identify its junction with the cystic duct and the common hepatic duct. The common hepatic duct was followed proximally to its union with the right and left hepatic ducts at the porta hepatis [11]. The hepatic artery proper was then carefully dissected free of surrounding connective tissue and autonomic plexus to allow full visualization of its course and branching pattern [11].

Additionally, the right gastric artery was identified arising from the hepatic artery proper and traced toward the lesser curvature of the stomach. The hepatic portal vein, lying posterior to the bile duct and hepatic artery proper, was dissected and followed both superiorly to its bifurcation at the porta hepatis and inferiorly behind the first part of the duodenum [11]. Tributaries from the left and right gastric veins were noted entering the portal vein along its course [11].

Visible lymph nodes within the hepatoduodenal ligament were identified and excised [11]. Due to the limitations of embalming, lymphatic vessels were not visualized and were not pursued. All anatomical structures, including both arterial variations, were cleaned and preserved in situ for documentation. Photographs were taken throughout the procedure to support the written observations. The concurrent presence of Moynihan’s hump and a double cystic artery was noted as a rare anatomical finding with potential clinical implications during hepatobiliary surgery.

**Figure 1:**
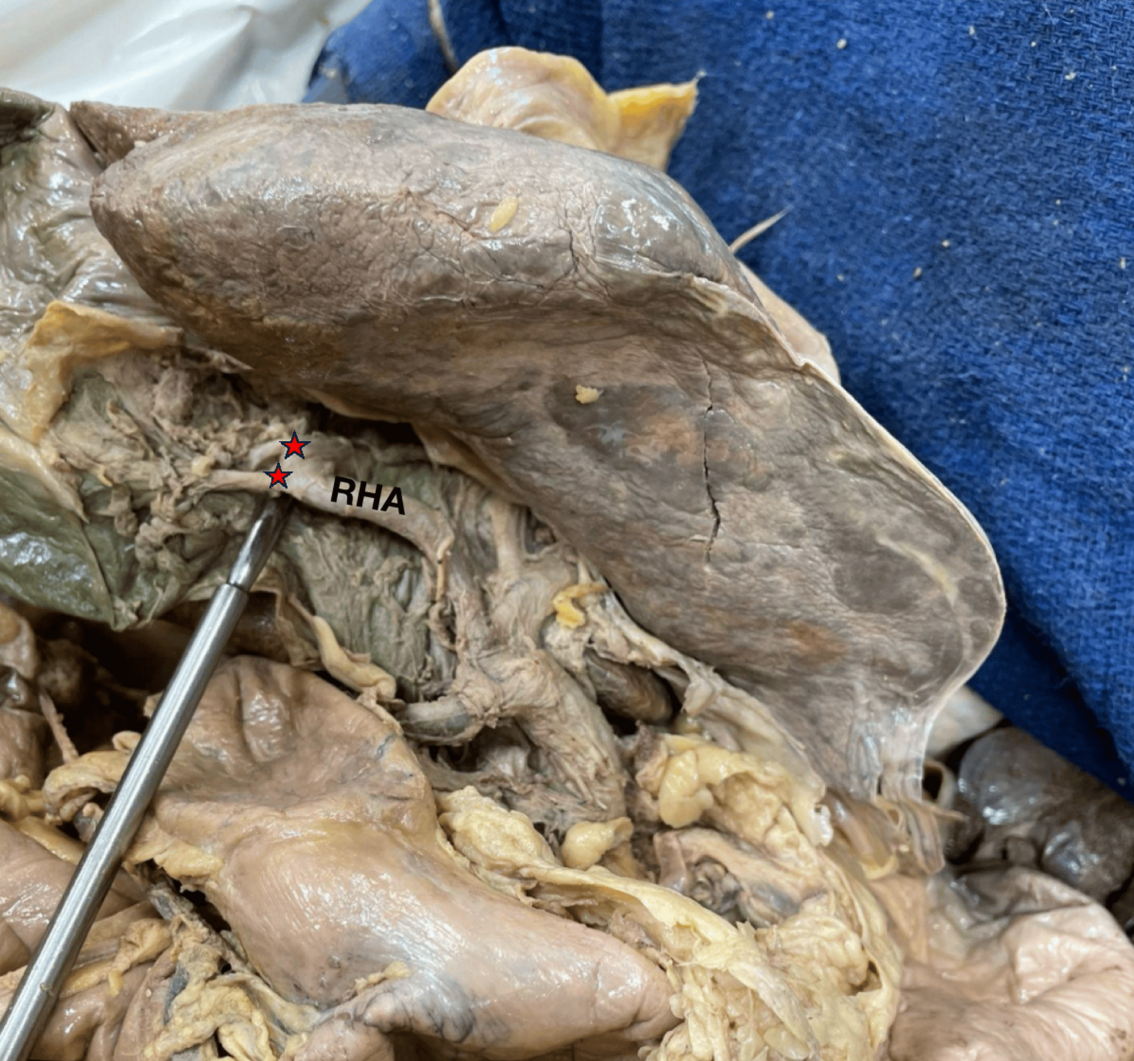
Double cystic arteries arising from a Moynihan’s hump of the right hepatic artery. The red stars indicate two cystic arteries arising from the right hepatic artery (RHA). Each cystic artery lies laterally to the stars. Additionally, these arteries appear to arise from Moynihan's hump, as suggested by the tortuous path the right hepatic artery takes to supply the right lobe of the liver in conjunction with noticeably short cystic arteries.

During dissection, two significant anatomical variations were identified. First, the RHA was found to take a tortuous course, looping anterior to the common bile duct within the hepatoduodenal ligament, consistent with Moynihan’s hump. This artery was traced proximally to its origin from the hepatic artery proper and distally to its entry into the liver parenchyma. Second, the presence of a double cystic artery was observed. Both arteries arose independently from the RHA near the hump and entered the gallbladder through Calot’s triangle. The anterior branch coursed along the cystic duct, while the posterior branch entered the body of the gallbladder directly. The coexistence of Moynihan’s hump with duplicated cystic arteries significantly increased the arterial complexity within Calot’s triangle (Figure 1). 

## Discussion

Current surgical guidelines emphasize achieving the Critical View of Safety (CVS) to minimize intraoperative errors and prevent misidentification of key structures [10-13]. Obtaining CVS depends on the surgeon’s ability to correctly identify Calot’s triangle and recognize anatomical variations within it. However, Moynihan’s hump can obscure normal anatomical relationships, making it more difficult to establish CVS and increasing the risk of misidentification and vascular injury. A systematic review of laparoscopic cholecystectomies found that vascular anomalies within the hepatobiliary triangle contribute to surgical complications, with Moynihan’s hump posing a specific risk for inadvertent ligation [13].

Recognizing these challenges, current literature strongly advocates for the early implementation of a complementary anatomical curriculum highlighting common variations that may be encountered during surgery in an effort to reduce future surgical errors [12-15]. However, despite the well-established impact of anatomical variation on surgical outcomes, medical curricula have not consistently integrated this knowledge in a structured and clinically relevant manner [12-15]. A survey of US medical schools found that while 88.6% of programs acknowledged the importance of teaching anatomical variations during preclinical education, fewer than half had instituted methods to assess students’ knowledge of these anomalies [12]. This lack of formal evaluation creates knowledge gaps that persist into postgraduate training, leaving new surgeons underprepared to recognize and manage anatomical deviations [14].

The lack of exposure to anatomical variations during training has broader implications for patient safety. Studies indicate that inadequate exposure to anatomical variation contributes significantly to surgical errors, with an estimated 25% of malpractice claims in both general and vascular surgery being linked to an inability to recognize anatomical anomalies [14]. These findings highlight the need for curriculum enhancement that prioritizes real-world anatomical variability in early medical education. To address these deficiencies, researchers have proposed several strategies to bridge the gap between anatomical education and clinical application, including (1) cadaveric dissection and prosection, (2) pre-dissection imaging and radiological integration, (3) simulation-based learning, (4) digital archiving and Universal Design for Learning (UDL), and (5) assessment and curriculum reform [12-15].

**Figure 2:**
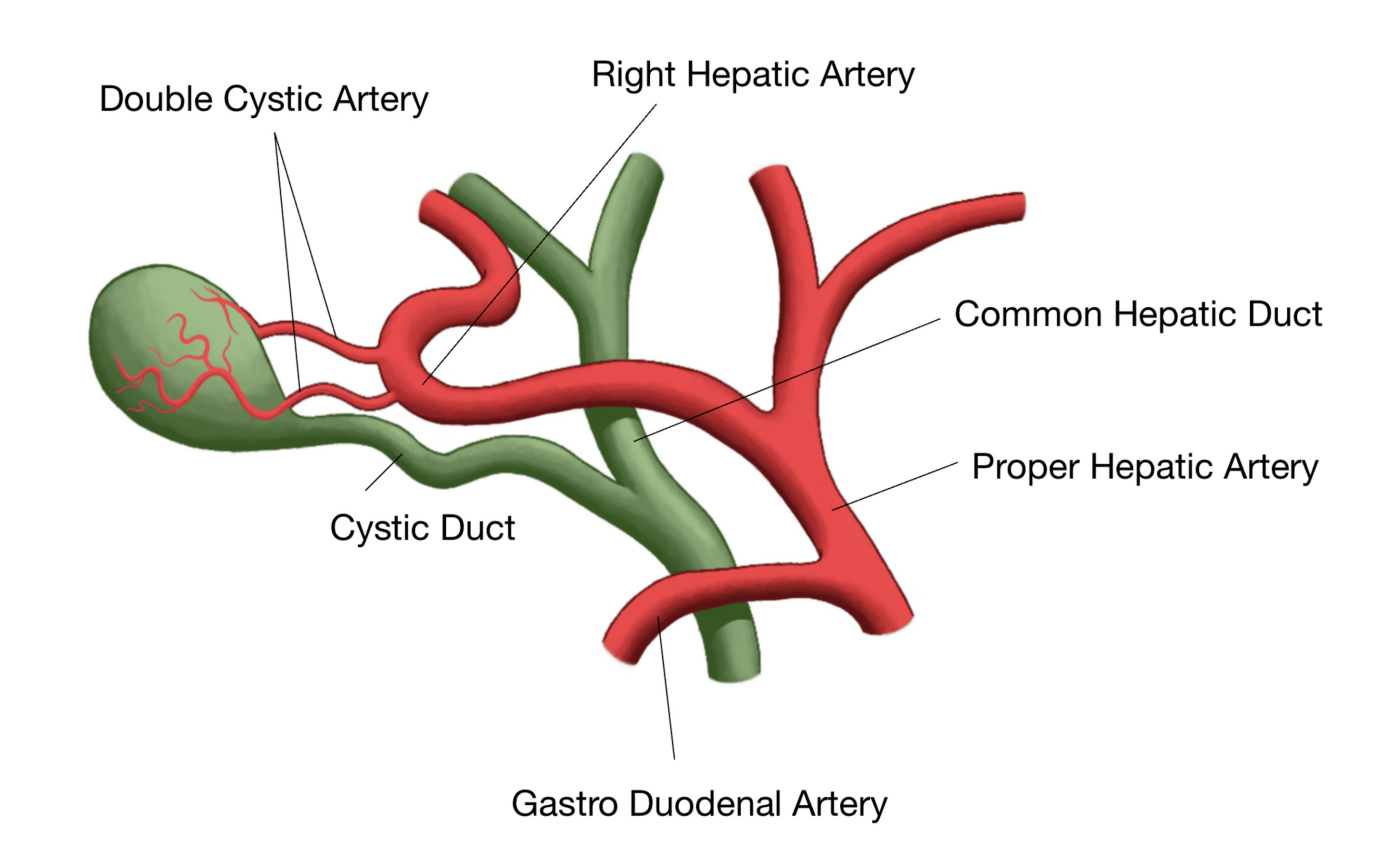
Original illustration depicting Moynihan’s hump of the right hepatic artery giving rise to two cystic arteries. This original anatomical illustration was derived from cadaveric findings, showing Moynihan’s hump of the right hepatic artery giving rise to two cystic arteries. This serves as an example of pre-dissection imaging that can aid in anticipating surgical complexity and preventing vascular injury. Image Credits: Uchenna Uduma. This image was created using the Notability app (Ginger Labs, Inc., San Francisco, California).

To address this educational gap, we developed an original anatomical illustration derived from the cadaveric findings to serve as a form of pre-dissection imaging, as depicted in Figure 2. 

This visual model translates complex vascular relationships into a format suitable for pre-lab preparation, surgical simulation, and anatomy instruction. By presenting Moynihan’s hump and dual cystic arteries in a clear schematic context, the illustration bridges the gap between discovery and application, transforming a rare cadaveric variation into a teaching tool that enhances anatomical literacy and promotes patient safety through improved surgical orientation.

## Conclusions

The present study contributes to the expanding knowledge of hepatobiliary vascular anomalies and is clinically significant, as familiarity with variations within Calot’s triangle is essential for preventing intraoperative complications. In this case, the presence of a Moynihan’s hump was associated with dual cystic arteries. This study introduces a new anatomical illustration depicting the relationship between the tortuous RHA and the duplicated cystic arteries, providing a clear visual representation that may aid in preoperative planning, surgical education, and anatomical instruction. By combining detailed cadaveric findings with updated anatomical depictions, this case report offers a valuable resource for recognizing Moynihan’s hump in conjunction with dual cystic arteries.

## References

[1] Agur AM, Dalley AF II (2025). Grant’s Atlas of Anatomy. https://internationalanatomy.lwwhealthlibrary.com/book.aspx?bookid=3319.

[2] Andall RG, Matusz P, du Plessis M, Ward R, Tubbs RS, Loukas M (2016). The clinical anatomy of cystic artery variations: a review of over 9800 cases. Surg Radiol Anat.

[3] Asghar A, Priya A, Patra A, Gupta P, Kumar A (2023). Moynihan's hump of the right hepatic artery in Calot's triangle: a systematic review and meta-analysis of its incidence and surgical importance. Surg Radiol Anat.

[4] Kamath.B K (2015). Dual cystic arteries in association with caterpillar hump of right hepatic artery- a case report and its surgical relevance. J Clin Diagn Res.

[5] Dalley AF II, Agur AM (2023). Moore’s Clinically Oriented Anatomy. https://shop.lww.com/moore-s-clinically-oriented-anatomy/p/9781975209544?srsltid=AfmBOoqt4AKSS8cuUf36Z4V4ox5K6D9DilZKkPdA4oaxKuHWn5Mo6IK1.

[6] Marano L, Bartoli A, Polom K, Bellochi R, Spaziani A, Castagnoli G (2019). The unwanted third wheel in the Calot's triangle. Incidence and surgical significance of caterpillar hump of right hepatic artery with a systematic review of the literature. J Minim Access Surg.

[7] Martín Pérez JA, Domínguez Rodríguez JA, De Alba Cruz I, Lara Valdés AJ, Sánchez Baltazar AL, Perna Lozada L (2021). Moynihan's Lump as an unusual variant of right hepatic artery during a laparoscopic cholecystectomy approach. A case report. Int J Surg Case Rep.

[8] Nagral S (2005). Anatomy relevant to cholecystectomy. J Minim Access Surg.

[9] Singh K, Singh R, Kaur M (2017). Clinical reappraisal of vasculobiliary anatomy relevant to laparoscopic cholecystectomy. J Minim Access Surg.

[10] Uhe I, Ghyasi AG, Chevallay M, Cherbanyk F (2022). A 56-year-old woman with acute cholecystitis and a Moynihan's hump, or caterpillar configuration, of the right hepatic artery identified during laparoscopic cholecystectomy. Am J Case Rep.

[11] Detton AJ (2021). Grant’s Dissector. https://pa-core.lwwhealthlibrary.com/book.aspx?bookid=2832.

[12] McClellan K, Crossley K (2024). Anatomic variation used to assess clinical reasoning. IMJ Translational Med.

[13] Asghar A, Patra A, Naaz S, Kumar R, Babu CS, Singh B (2024). Investigating the integration of anatomical variabilities into medical education as a potential strategy for mitigating surgical errors. J Anat Soc India.

[14] Kowalczyk KA, Majewski A (2021). Analysis of surgical errors associated with anatomical variations clinically relevant in general surgery. Review of the literature. Transl Res Anat.

[15] Cullinane DP, Barry DS (2022). Breaking the norm: anatomical variation and its key role in medical education. Anat Sci Educ.

